# Subacute Combined Degeneration of the Spinal Cord due to Different Etiologies and Improvement of MRI Findings

**DOI:** 10.1155/2013/159649

**Published:** 2013-03-27

**Authors:** Azize Esra Gürsoy, Mehmet Kolukısa, Gülsen Babacan-Yıldız, Arif Çelebi

**Affiliations:** Department of Neurology, Medical Faculty, Bezmialem Vakıf University, Turkey

## Abstract

Subacute combined degeneration (SCD) is a rare neurological complication of vitamin B12 deficiency, characterized by demyelination of the dorsal and lateral spinal cord. Herein, we describe three cases, who presented with SCD, one related to reduced intake of vitamin B12 because of a vegetarian diet and two related to nitrous oxide exposure during surgery. MR images of our patients revealed symmetrical hyperintense signals in dorsal and lateral columns in T2 weighted series. After treatment with intramuscular B12 injections (1 mg daily for 2 weeks, once weekly thereafter for three months) all patients showed improvement of their symptoms. Abnormalities of the spinal cord on MRI resolved in three months. In conclusion, SCD either due to nitrous oxide exposure or due to reduced intake of vitamin B12 is a reversible condition, when detected and treated early.

## 1. Introduction

Vitamin B12 deficiency causes a wide range of hematological, gastrointestinal, and neuropsychiatric disorders [1]. Myelopathy, neuropathy, dementia, behavioral changes, and optic nerve involvement are common neurologic manifestations of vitamin B12 deficiency [1–3]. The most frequent causes of vitamin B12 deficiency are malabsorption (e.g., pernicious anaemia, gastrectomy, intestinal infections, tropical sprue, terminal ileal conditions including Crohn's disease, and resection), pancreatic exocrine insufficiency, medications (colchicine, neomycin, and p-aminosalicylic acid), disorders of intracellular cobalamin metabolism (methylmalonic aciduria and homocystinuria), increased requirement (in hyperthyroidism and alpha thalassemia), and inadequate intake with food (e.g., vegetarianism and veganism) [1–3]. Nitrous oxide exposure during surgical procedures or recreational use is a rare cause of acute vitamin B12 inactivation [4–6]. The spinal cord lesion caused by vitamin B12 deficiency is known as subacute combined degeneration (SCD). Here we describe three patients, who presented with SCD, one related to reduced intake of vitamin B12 because of a vegetarian diet and two related to nitrous oxide exposure during surgery, with improvement both clinical and radiological after vitamin B12 replacement.

## 2. Case Reports

### 2.1. Case 1

**Figure 1:**
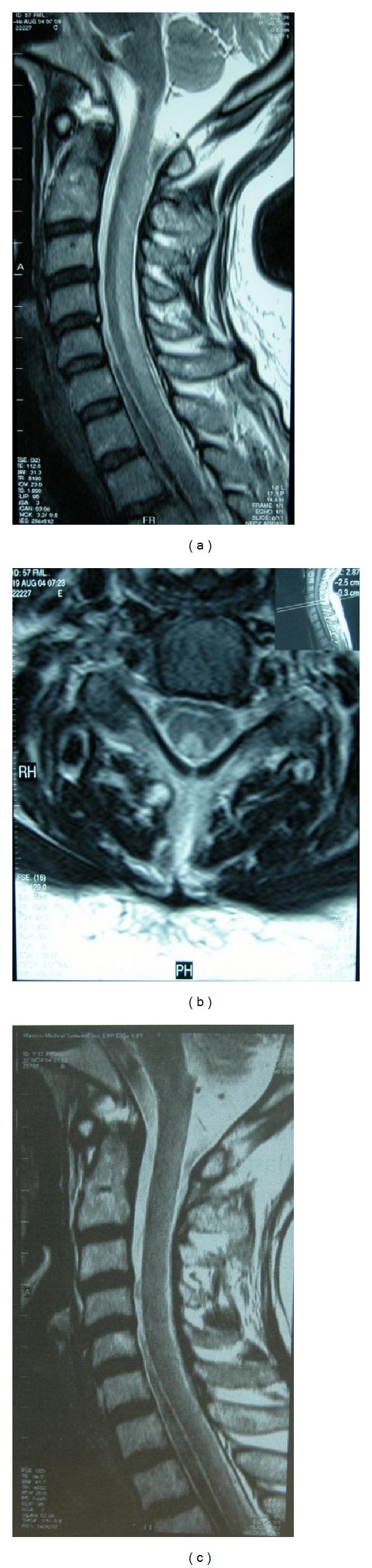
((a), (b)) At the baseline sagittal and axial T2 weighted MRI study, hyperintensity of the posterior columns can be seen in the cervical cord. (c) Posttreatment sagittal T2 magnetic resonance images showed total regression of the MRI abnormalities.

A 58-year-old female patient presented with gradually worsening unsteady gait as well as numbness and tingling in hands and feet. She had undergone thyroidectomy operation 6 weeks before her admission, for which she had 60 minutes general anesthesia with nitrous oxide. At the time of admission neurological examination revealed slight weakness of distal muscles, bilaterally absent ankle jerk reflexes, bilaterally extensor plantar reflexes, and loss of position and vibration senses in all extremities. She could walk with assistance and Romberg's sign was positive. Memory and other cognitive functions were preserved. Laboratory tests showed no anemia (hemoglobin: 12.4 g/dL; normal range 11.5–15.5 g/dL) but an increased mean corpuscular volume (MCV) (103 fL; normal range 80–100 fL). The level of vitamin B12 was low (143 pg/mL; normal > 211 pg/mL) while folate and serum copper levels were normal. Cerebrospinal fluid (CSF) examination was normal. Brain MRI was unremarkable. T2 weighted MRI of the cervical spinal cord showed high signal lesions in the posterior columns extending to the upper thoracic spinal cord (Figures 1(a) and 1(b)). Nerve conduction studies revealed sensorial axonal polyneuropathy. A diagnosis of SCD of the spinal cord and axonal polyneuropathy related to nitrous oxide anesthesia were established. Parenteral vitamin B12 therapy was immediately started with 1 mg daily injections for 2 weeks and once weekly thereafter. At three-month followup the patient was able to walk unassisted and strength was normal in all four limbs, whereas vibratory sense was impaired. There was a total regression of the MRI abnormalities described previously (Figure 1(c)).

### 2.2. Case 2

**Figure 2:**
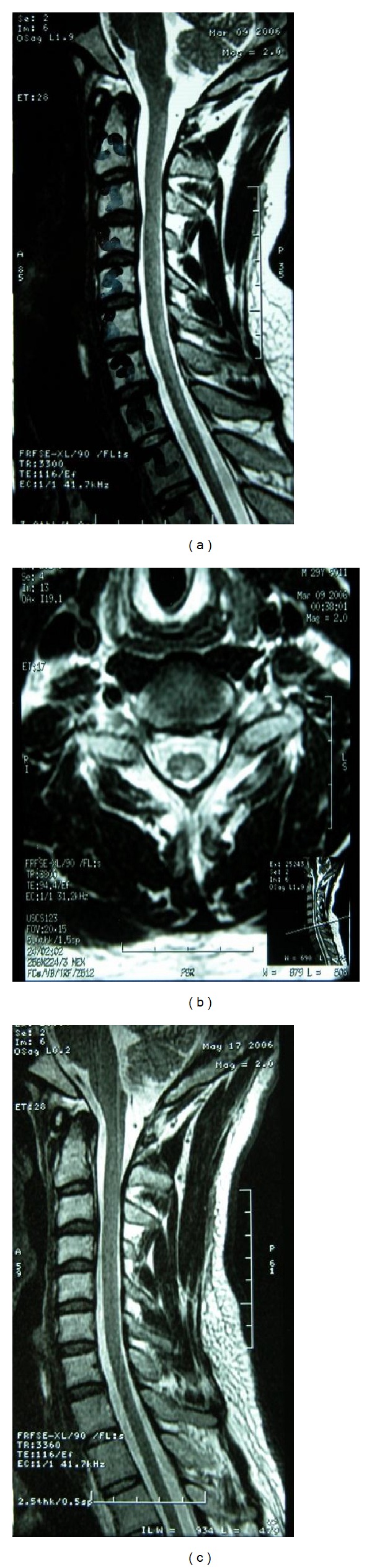
((a), (b)) Sagittal and axial T2 magnetic resonance images on patient admission showed T2 hyperintensity of the posterior columns. (c) Sagittal T2 magnetic resonance images 2 months later showed striking reduction of the MRI abnormalities.

A 28-year-old man was admitted to the hospital with gait imbalance, unsteadiness, and numbness in hands and feet. He had a history of a generalized tonic-clonic seizure 6 weeks ago and his cranial MR images showed agenesis of the corpus callosum and a cystic lesion in the interhemispheric fissure. He had undergone ventriculoperitoneal shunting under nitrous oxide anesthesia 5 weeks before his admission. One month after the operation gait imbalance, unsteadiness and paresthesia were developed. Neurological examination revealed slight left hemiparesis and symmetrically increased patellar and ankle reflexes. Plantar reflexes were extensor. There was complete absence of vibration and proprioception senses in the lower limbs and impairment in the upper limbs. Romberg's sign was positive. There was no sphincter dysfunction. Memory and other cognitive functions were preserved. Laboratory findings showed macrocytic anemia (hemoglobin: 9.9 g/dL and MCV: 101.3 fL) and decreased vitamin B12 level (<100 pg/mL). MRI of cervical and thoracic spinal cord showed symmetrical hyperintense signal changes on T2 weighted images of the posterior columns at C6-D3 segments (Figures 2(a) and 2(b)). A diagnosis of SCD of the spinal cord related to nitrous oxide anesthesia was established. Parenteral cobalamin therapy was immediately started with 1 mg daily injections for 14 days and once weekly thereafter. Two months after the onset of administration of parenteral vitamin B12 therapy, MRI of the cord showed striking reduction of the posterior column changes (Figure 2(c)). Clinical neurological findings resolved completely after 6 months.

### 2.3. Case 3

**Figure 3:**
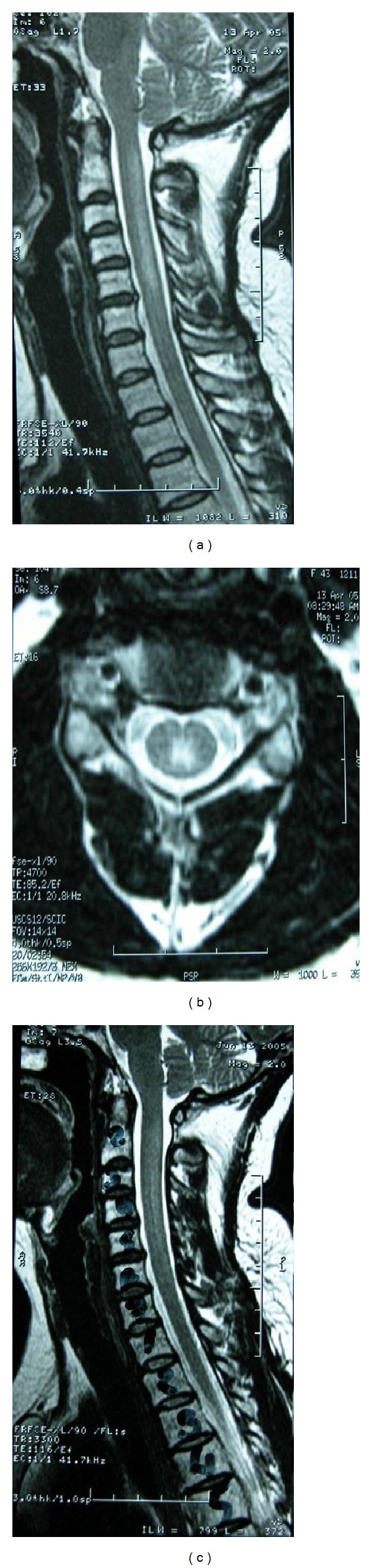
((a), (b)) Sagittal and axial T2 weighted images of the cervical cord before treatment demonstrate high signal bilaterally in the posterior columns of the cord. (c) The posttreatment images 2 months later show a striking reduction of the MRI abnormalities.

A 44-year-old female patient presented with gradually worsening paresthesia and tingling of both hands and feet for 6 weeks. She had been taking a vegetarian diet for nine months. On neurological examination there was slight paresis of both hands. Deep tendon reflexes were increased and plantar reflexes were flexor. Proprioception and vibration senses were impaired in both hands and feet. Romberg's sign was positive. She denied any bladder or bowel dysfunction. Memory and other cognitive functions were preserved. Laboratory investigations revealed macrocytosis but no anemia (hemoglobin: 13.5 g/dL and MCV: 110 fL). The serum vitamin B12 level was 135 pg/mL. Serum copper was 106 *μ*g/dL (normal range 80–155 *μ*g/dL). An MRI study of the cervical spine showed hyperintensities of the posterior columns in T2 weighted sequences (Figures 3(a) and 3(b)). A diagnosis of SCD of the spinal cord related to inadequate intake of vitamin B12 was established. Parenteral vitamin B12 therapy was started 1 mg daily for 2 weeks and once weekly thereafter. Complete clinical improvement occurred during two months and two months later there was a striking reduction of the MRI abnormalities (Figure 3(c)).

## 3. Discussion

Vitamin B12 deficiency is a systemic disease that often affects the nervous and hematological systems. Megaloblastic anemia is a common early manifestation pointing to an underlying vitamin B12 deficiency, although neurological symptoms may occur in the absence of hematological abnormalities [2]. Although all of our patients had increased MCV, only one of them had anemia. Neurologic symptoms of vitamin B12 deficiency are paresthesias, diminished proprioception and vibration sensation, motor weakness, clonus or hyperreflexia, areflexia, autonomic dysfunction, gait disturbance, intellectual or behavioral impairment, and impaired visual acuity [3]. The most frequent neurologic manifestations are the SCD of the spinal cord and polyneuropathy [1, 3]. SCD affects the posterior columns and the corticospinal tracts and is characterized by swelling of the myelin sheaths and a patchy myelopathic spongy vacuolation of the affected regions of the cord [6, 7]. 

**Figure 4:**
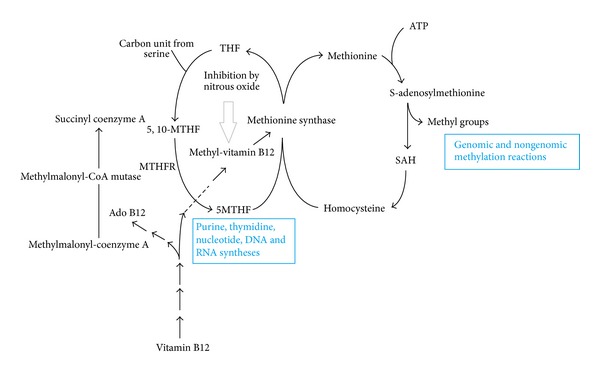
Pathways of intracellular vitamin B12 metabolism. MTHFR: methylenetetrahydrofolate reductase; 5,10-MTHF: 5,10-methylenetetrahydrofolate; 5-MTHF: 5-methylenetetrahydrofolate; THF: tetrahydrofolate; SAH: S-adenosyl homocysteine.

Methyl-vitamin B12 (Met B12) and 5′-deoxy-5′-adenosylcobalamin (Ado B12) are two physiologically active forms of vitamin B12 in the human body [8]. Met B12 is needed as a cofactor by the methyltetrahydrofolate-homocysteine methyltransferase (MTR) enzyme to generate methionine from homocysteine and tetrahydrofolate from methyltetrahydrofolate. Ado B12 is required as a cofactor by the methylmalonyl-CoA mutase (MMCoAM) for the conversion of methylmalonyl-coenzyme A to succinyl coenzyme A [8]. Hypotheses proposed for the development of SCD are still controversial. The Ado B12-MMCoAM hypothesis was the leading one in terms of the biochemical basis of B12 deficiency myelopathy. The dysfunction of the AdoB12-MMCoAM pathway leads to an intracellular accumulation of both propionyl-CoA and methylmalonyl-CoA leading to the formation of abnormal fatty acids. A strong evidence against the AdoB12-MMCoAM pathway hypothesis is nitrous oxide induced SCD because it is known that nitrous oxide exposure affects primarily the Met B12-MTR pathway without initially affecting the AdoB12-MMCoAM pathway. Nitrous oxide inactivates Met B12 by irreversibly oxidizing the cobalt core (Figure 4) [9]. Methionine is the precursor of S-adenosylmethionine, a universal methyl donor which is important in the methylation of myelin basic protein and myelin lipids. Decreased methionine and S-adenosylmethionine lead to instability of the myelin sheath. Tetrahydrofolate is necessary for *de novo* DNA synthesis, and failure of the tetrahydrofolate pathway affects cells which undergo rapid turnover, such as blood cells. In the last decade there has been growing evidence of the imbalance in cytokines and growth factors related to B12 deficiency leading to SCD. In the gastrectomized rat model, vitamin B12 deficiency leads to increased spinal cord synthesis and increased cerebrospinal fluid (CSF) levels of two myelinotoxic cytokines, tumor necrosis factor-alpha (TNF-*α*) and soluble (s) CD40:sCD40 ligand dyad, and a myelinotoxic growth factor, nerve growth factor (NGF). Conversely, B12 deficiency decreases spinal cord synthesis and CSF levels of a myelinotrophic cytokine, interleukin 6 (IL-6), and a myelinotrophic growth factor, epidermal growth factor (EGF) [10]. 

MRI findings in SCD can be diagnostically extremely helpful. MRI shows a very typical pattern with T2 hyperintense signal alterations usually confined to the posterior columns, which may involve the lateral columns and rarely brainstem [11]. Cervical spinal MRI showed in all of our cases typical symmetrical T2 signal hyperintensities in posterior columns but no contrast enhancement. Symmetrical hyperintense signals in lateral columns and posterior columns in diffusion weighted imaging (DWI) have been reported [12, 13]. MRI seems to be a good diagnostic tool for the follow-up evaluation. Vasconcelos et al. concluded that the absence of sensory dermatomal deficit, Romberg, and Babinski signs, MRI lesions in ≤7 segments, and age younger than 50 were associated with a higher complete resolution rate [14]. Similarly our two patients younger than 50 years improved completely. However, we do not think that Romberg sign is a prognostic factor in SCD, because we revealed Romberg sign in all of our patients including patients who improved completely. The cord abnormality can resolve without evidence of cord atrophy on MRI, if treated early. As previously described, resolution of spinal lesions on MRI might precede the clinical improvement by several months [15]. However, diagnostic delay and/or late initiation of therapy may result in permanent irreversible injury to the spinal cord with little or no improvement on treatment. Our cases were diagnosed early and clinical and radiological improvements were observed during followup.

Ataxic myelopathy might be seen in patients with copper deficiency. Copper deficiency myelopathy is associated with symmetric involvement of the pyramidal tract and posterior columns, and it shares same clinical and radiologic pictures with SCD. Therefore copper deficiency should be kept in mind in the differential diagnosis in patients with ataxic myelopathy. Malnutrition, prematurity, parenteral or enteral feeding without copper supplementation, gastrectomy, ingestion of copper-chelating agents, and excessive zinc therapy are among the most common causes of copper deficiency [16]. Two of our patients had normal serum copper levels. Copper deficiency myelopathy was also excluded in the second patient, because of improvement of symptoms and reduction of MRI abnormalities after vitamin B12 replacement therapy.

In conclusion a prompt diagnosis of SCD and early vitamin B12 treatment could avoid irreversible neurologic damages and prevent disability.
